# On Epilepsy

**Published:** 1855-04-01

**Authors:** 


### Art. IV.—ON EPILEPSY.

(*Continued from page* 51.)

VII. *How is Epilepsy distinguished from other Affections?*—The characters of epilepsy are in general so well marked and distinctive that there is no difficulty in the diagnosis. It may be confounded with eclampsia, of which it may be considered a chronic form—its termination, or recurrence as a chronic affection, will sufficiently distinguish it. From hysteria, epilepsy is distinguished by the total loss of consciousness during the attack, which is never met with in hysterical patients, and by the more *regular* convulsive action in hysteria, by its mode of accession, and by the circumstances as to age, sex, &c., of the patient.

In apoplexy, there is little or no convulsion, and though there is complete unconsciousness, there is none of that embarrassment of the circulation and respiration, which is so characteristic of the epileptic seizure. For the distinction between epilepsy and the simulated affection, we refer to systematic writers on the subject; sensibility to pain, or the action of the pupil when exposed to sudden light, will in general be sufficient tests.

VIII. *Prognosis.*—From what has been already stated, it will readily be concluded that the prognosis of epilepsy is most serious, and fully justifies the forcible remarks of Georget: "Epilepsy is one of the most horrible of diseases, it shortens life, it kills occasionally in one attack, it finishes ordinarily by degrading and annihilating the intellect, it excludes the sufferer from society, and renders his life insupportable." The opinions given upon its curability are most strikingly at variance with each other, even from the highest authorities. We will quote a few of these by way of illustration.

Hippocrates recognises its curability under these limitations:—"Whoever is acquainted with such a change in men, and can render a man humid and dry, hot and cold by regimen, could also cure this disease, if he recognises the proper season for administering his remedies." Aretæus considers that when once firmly fixed and rooted in the system, it lives with the patient, and only dies with him.

Paulus Ægineta considers it susceptible of cure, without drawing the distinction between centric and excentric epilepsy. Of moderns, Maisonneuve considers its resistance to treatment almost insurmountable; Pinel gives a guarded and doubtful opinion; Hufeland says:—"the prognosis is sad, and the disease difficult to cure,—the curability is in the proportion of one to twenty—the immediate mortality is rare." Chomel considers medicine as "almost always powerless" in curing epilepsy. It is proper to observe here, that these remarks refer chiefly to centric or cerebral epilepsy. Portal considers epilepsy one of those diseases which is the most rarely cured, but that more success attends the treatment than is generally supposed. M. Foville concludes that by a careful attention to causes, great success might be hoped for in a majority of cases. M. Esquirol, a very high authority, after stating his endeavours at the Salpêtrière, and their occasional results in suspending the attacks, adds :—

" *Plusieurs de nos épileptiques se sont pretées à mes essais plusieurs années ; mais, l'avouerai-je ! je n'ai pu obtenir de guérison.*"

He considers the suspension of the attacks in most cases to be due to the mental excitement of trying new means of cure or of consulting a new physician, and concludes that epilepsy is rarely curable. Of the reported cures, he considers some to be hysteria, and others to be mere suspensions of the attacks, the patients being lost sight of.

Henry asserts that the cure of epilepsy ought to be regarded as an extremely rare exception, and that epilepsy complicated with insanity is never cured.

M. Lelut, at the Salpêtrière, and M. Delasiauve, at the Bicêtre, disbelieve altogether in the curability of epilepsy, and consider the attempt as "le desespoir de la médecine."

On the other hand M. De la Rive thinks epilepsy curable by nitrate of silver in most cases, whilst M. Foville speaks in terms of the strongest reprobation of it; and M. Debreyne thinks it curable by extract of belladonna, whilst others of high authority consider it quite useless.

M. Herpin gives the particulars of sixty-eight cases analysed with great care, from which he draws the following inferences as to the general prognosis :—

1. That epilepsy is not generally spontaneously cured by the efforts of nature alone, though this may occur in one-twentieth of the cases observed.

2. That treatment appears to produce a beneficial result in three-fourths of those treated: that a cure may be expected in half, and great amelioration in about one-fifth.

3. That about one-fourth of the cases are incurable.

This summary would be more valuable, if the distinction we have so frequently mentioned had been kept in mind—viz., between the centric or cerebral and the excentric or symptomatic epilepsy. How are we to reconcile such contradictory evidence ? and to what conclusions are we to come ?

For the extremely unfavourable judgment given by Monneret and Fleury, Delasiauve, Esquirol, and Lelut, we may partly account, inasmuch as their experience was probably chiefly amongst old and confirmed cases, and such as were complicated in most instances with mental affections, at the Salpêtrière and Bicêtre — and such cases, we have before stated to be nearly if not altogether incurable. It is more than probable that all these would be cases of epilepsia centrica vel cerebralis.

On the other hand, M. Herpin's calculations, and those of many of the others, are founded upon private experience, or the practice of such hospitals as receive cases more promiscuously than the above, and where there would consequently be a great proportion of the more frequently occurring cases, those of epilepsia eccentrica vel sympathetica; and in this we have a key to the varying accounts.

A reconsideration of the phenomena will assist us again in forming a rational prognosis. A person of 20 years old, in the enjoyment of apparently perfect health, is seized with a series of epileptic fits, but in the intervals, the health is good, the intellect sound and vigorous, the muscular system unimpaired, and the functions generally performed properly; and we can trace no deformity of cranium, or other indication of malformation. We conclude, or hope to find, this to be a case of excentric or symptomatic epilepsy, dependent upon some functional disorder; and, seeing that in such a subject, there are times when the functions are so co-ordinated as to be compatible with perfect health, we have reason to hope that by a judicious use of hygienic and therapeutic agents, this state may be attained and permanently preserved. And as a general rule, we may state with regard to symptomatic epilepsy, that it is curable in so far as the source of irritation is capable of relief or removal, and in proportion to the care and skill brought to bear upon the exciting functional disorder. And thus we may hope, with care, to cure the majority of such cases, and to benefit many more. And what of the centric or cerebral epilepsy? The same general rule of prognosis will apply, but with restrictions. There is evidently a condition of the functions compatible with health; and a careful observation of the precise energy of each function *during health*, combined with judicious attempts to preserve them in that state, will not fail greatly to diminish the number and violence of the attacks, even though perfect success may not attend our efforts. So far these remarks apply to the general prognosis of the disease; when we wish to form an opinion upon any individual case, we have many other circumstances to take into account.

The hereditary tendency to the disease is an unfavourable sign, as indicating the probability of its being E. centrica. *Sex* appears to exert some little influence upon the curability of epilepsy—women are more liable to the attack, but more easily cured. The most favourable age for the first attack, as regards curability, is from 10 to 20. Long duration of the disease is evidently conducive to an unfavourable judgment. M. Herpin holds an opinion very strongly, that the curability or obstinacy of epilepsy may be predicated with considerable certainty from the number of attacks already experienced. We give his conclusions, but offer no opinion upon them:—

"A cure may be assured almost certainly to patients who have only the 'petit mal,' who have it not very frequently, and with whom it has not lasted above 10 years.

"In the ordinary attacks the prognosis is *favourable* if they have not exceeded 100.

"The chances of success are less between 100 and 500.

"The prognosis is extremely unfavourable when the attacks have exceeded 500."

We cannot conclude this branch of the subject without referring to the aphorisms of M. Esquirol on the subject of epilepsy:—

1. "Epilepsy is a long and dangerous disease; rarely fatal in the first attack." 2. "When hereditary, it is rarely curable." 3. "Symptomatic epilepsy is more easily cured than the centric form, though this last is not always incurable." 4. "Sometimes epilepsy disappears for many years, to re-appear without any known cause." 5. "Those attacked soon after birth, are rarely cured; if puberty has not the effect, they remain incurable." 6. "Those attacked between 3 and 4 years are generally curable, if treated in time." 7. "Those attacked just before puberty are cured on the completion of the crisis." 8. "Marriage only cures genital epilepsy; it aggravates other species." 9. "A pregnant woman, who becomes epileptic, runs very great risk." 10. "When the crises increase in frequency and intensity, death is probably imminent." 11. "Death does not take place during the horror of the convulsions, but the period of the depression afterwards." 12. "Epilepsy complicated with mental alienation *is never cured*."

IX. *The Treatment of Epilepsy.*—Fifty years ago, it appears that the remedies for epilepsy were so numerous as to occupy 150 quarto pages of the "Analecta Literaria Epilepsiam Spectantia,"—they have since multiplied — it is utterly impossible to give even the briefest analysis of them. One author vaunts belladonna as almost invariably successful; another, after a fair trial, pronounces it worthless. The same is the case with valerian. Dr. Heim pronounces *nitrate of silver* to be the most effectual remedy he has found in an extensive practice of sixty years. M. Herpin upholds *oxyde of zinc* as the most valuable of all remedies. M. Foville speaks in terms of the *strongest reprobation* of the use of *both* these remedial agents, and all *metallic* oxydes.

The weight of testimony amongst reputable authors is quite against the use of *specifics*; and indeed, it seems but rational to suppose, that if epilepsy depends upon a disorder of the stomach, it will not be materially benefited by valerian,—or if upon the liver, that nitrate of silver will not be of service, and so for the rest.

In default of a rational system of treatment, the most extraordinary and heterogeneous matters have been forced upon the unfortunate stomachs of the already sufficiently unfortunate patients—they are too disgusting even to enumerate. After a short catalogue of these, Dr. Cheyne remarks:—

"There are other abominations of the same kind, unnecessary to specify, the use of which, Erastus alleges, was taught to mankind by the devil; but without calling in question the active malignity of our great enemy, we are of opinion that man, when left to his own inventions, is fully equal to the discovery of these and a multitude of other therapeutic agents of equal ineptitude." (Cyc. of Pract. Med., Art. Epilepsy.)

We believe that the true principles of treatment are to be sought, as we have indicated under the head of prognosis, in observation of that state of co-ordination of the functions which is compatible with health during the intervals—and in the attempt to preserve this co-ordination, whilst by the use of stimulants and tonics, we endeavour to raise the whole tone and energy of their performance.

Dr. Radcliffe's remarks on treatment indicate as strongly the eminently practical physician, as his investigations into the pathology of the affection show the philosopher and earnest searcher after truth. We can but refer our readers to his volume, our limits only permitting us to indicate the principles of treatment without details.

We must first aim at *correcting disordered function*—we must treat epilepsy according to its seat, when this is discoverable. For full details on this head, we refer to Dr. Cheyne's excellent article in the "Cyclopædia of Practical Medicine," more than once alluded to. The nutritive functions especially must be attended to, as to their regulation, and in reference to diet. On this last subject, as many differences of opinion exist as on the remedies to be employed. Fothergill, Abercrombie, and others of great weight, advise total abstinence from animal food and from all fermented liquor: Heberden relates cases cured by such means. Dr. Radcliffe, in accordance with that plan of treatment which has generally been found successful—viz., the tonic, advises abundant animal food, and a moderate allowance of stimulant. But all this must be regulated according to the condition and digestive powers of the patient—generally a mild, digestible, nutritious diet, with some form of stimulant, will be required. Exercise, short of fatigue, is always proper, and all hygienic methods in short, which will tend to the proper co-ordination of the functions. At the same time, we must attend to what has been very properly called, mental dietetics.

The influence of the mind is so strong as frequently to suspend the fits for some time—a new physician, or a new remedy has almost constantly this effect. It is only necessary to indicate this,—the particular method of application must suggest itself in the individual case. The precautions which must be taken in all cases against immediate personal injury during the fit, are too obvious to require notice.

We believe that by correcting disordered function, and by supporting the strength, and employing (but not over-employing) the mind, most cases of sympathetic epilepsy are curable, or susceptible of great relief. In the centric epilepsy, we must pursue the same system with, if possible, still more rigorous exactness,—but when these means fail, as they too frequently will, the temptation is irresistible to try some of the so-called specifics. The strongest, and perhaps in many instances the most effective, are the powerful mineral tonics—nitrate of silver, sulphate and ammonio-sulphate of copper, sulphate of zinc—these in almost incredible doses have been given, and apparently with good results; the oxyde of zinc in from one to five or ten grains, repeated from twice to six times daily, for weeks together, is spoken of with great favour by M. Herpin, and his results, fairly stated, are such as certainly recommend it to great attention. Of vegetable substances, the valerian, powdered root, in doses of from half a drachm to three drachms daily, is the most recommended. But on the subject of special remedies, we must refer to the various monographs and systematic works on epilepsy. We have little or no dependence on any treatment but such as is comprised in such indications as regulating disordered function—increasing tone and energy—and a proper attention to bodily and mental dietetics. Bleeding in any form is, as a rule, worse than useless. Setons, moxas, blisters, issues, may occasionally in old confirmed cases be productive of some relief, but we have not much to say in their favour.

But what of Dr. Marshall Hall's late panacea?

"With regard to tracheotomy," says, Dr. Radcliffe, "it is less easy to come to an opinion, and this the more as there is an insufficiency of evidence on the subject. Still it is clear that it does not fulfil all the original expectations of Dr. Marshall Hall concerning it. It does not prevent convulsion; it does not always, or even usually, make the convulsion slighter. It does not prevent danger; for of the few patients upon whom the operation has been performed, three have died either in the fit or in connexion with the fit; and of the three, the opening of the windpipe was free from all obstruction—at least in one. Under these circumstances, it becomes a question whether the benefits of the operation are sufficient to counterbalance the associated inconveniences and dangers even where (what rarely happens) the asphyxial symptoms are consequent upon spasmodic closure of the larynx; and this question must remain in abeyance for the present" (p. 133).

Now this is mincing the matter too much, for we hold that there is nothing in the pathology of the disorder, and scarcely more in the empiricism of experience, to justify the practice of making an additional vent-hole in the windpipe of the epileptic, seeing that, apart from the question of danger, the inevitable effect of this vent-hole, while it continues open, is to convert the possessor of it into "a dumb whistling wretch, whose every breath is an annoyance to himself and others." What is the real value of clinical evidence in this case has, we think, been shown by Dr. Radcliffe himself, in a paper read by him before the Medical Society of London, and reported in the *Lancet* for May 14, 1853; and we know of no new cases which require us to alter this opinion. These later cases may or may not be more favourable than the earlier, but one thing is certain—that one of their number—three or four in all—has died in the fit. For our own part, we have never been able to conquer the suspicion that all these cases, when their history is fully known, will turn out to be no more satisfactory than the two which are first in the series, and upon which Dr. Hall has dogmatized most loudly. We turn to Dr. Radcliffe's paper before mentioned for the particulars, and the natural comments upon them :—

"*Mr. Cane's Case.*—The patient was a boatman, aged twenty-four, who had been epileptic for seven or eight years. The fits were severe and frequent. The operation was performed during a fit, in consequence of a state of asphyxial coma that had lasted nineteen hours. The relief was immediate, and no fits have followed the operation. The habits of the patient were very irregular and intemperate, and he was discharged from his employment on this account about ten months ago. The tube is still worn, and curiously enough, it is worn with a cork in the opening.

"*Mr. Anderson's Case.*—The patient in this case was a stout, thick-set, muscular female, aged thirty-six, the daughter of an epileptic father, and herself epileptic for twenty-four years. Her complexion was ruined by the former use of nitrate of silver. The operation was performed in March, 1851, and the tube was worn until her death, which happened in a fit, about four months ago. After the operation the fits continued as before—possibly a little less frequently and severely, but decidedly of the same character. Her health and spirits also are said to have undergone some slight improvement, and she lost a numbness in her right arm, which had previously distressed her; but those who knew her best doubt the existence of any appreciable change of this kind until about two or three months before her death—sixteen months after the operation. The following notes of the final seizure are from Mr. Anderson :—'Eight A.M.: Had been up and dressed; heard to fall heavily. A woman removed the inner tube from the trachea, as she was in a fit apparently more severe than usual. She 'snorted loudly;' nails of a deeper colour. She was placed on the bed, as the woman thought she would recover as usual.' The woman here referred to says the patient was black in the face and violently convulsed, and that death must have taken place within ten minutes. The body was examined twenty-four hours after death, and the following are the particulars supplied by Mr. Anderson: 'Body extremely muscular; rigidity still present; not much fat. Head: vessels of scalp much congested; skull thick, and dura mater so universally adherent that the skull-cap could not be removed until the dura mater was divided. The sinuses were filled with dark blood, and on the removal of the brain an unusual quantity of dark blood flowed from the spinal canal. On either side of the longitudinal sinus, and on the inner side of the frontal bone, two or three growths of bone were found, and to these the dura mater was so fimrly adherent that on attempting to separate it, it was torn through and portions remained attached. The largest of the exostoses was about an inch and a half in circumference, and projected about half an inch from the surface of the bone. No alteration was observed in the corresponding portion of the cerebrum. The brain was softer than natural, and the puncta were more than usually distinct. There was little fluid in the ventricles, but the choroid plexuses were congested. Lungs: These organs were collapsed, occupying but little more than a third of the thoracic cavity, and somewhat congested at their posterior margin; structure healthy. Heart: Larger than usual (perhaps a fourth)'; cavities, especially the left, distended with blood; it was surrounded with fat, and its structure flabby; valves healthy. Liver, kidneys, and spleen, highly congested. Uterus natural, but cysts containing viscid fluid in the ovaries. Small intestines (especially lower part of the ilium) congested, and the mesenteric glands enlarged. Internal jugular, above the level of the omohyoid, almost empty." * * * * *

"What of Mr. Cane's case? Here undoubtedly the results seem most marked, but do they not prove too much? There are no fits whatever after the operation, and this is not to be expected, even on Dr. Hall's own premises. Moreover, fits do happen in all the other cases, and in some of them very severe fits, and this fact gives a probability of at least seven to one that the fits in this case did not keep away in consequence of the operation. It is to be remembered also that the wearing of the cork in the tracheal tube did, in fact, place the patient in the same predicament as that in which he was before the windpipe was opened. Why the fits kept away it is not necessary to inquire, for nothing is more certain than that epilepsy may suddenly disappear and keep away for a long time without any apparent cause.

"What of Mr. Anderson's case? Here the main questions are as to the character of the fits, the state of the general health, and the cause of death. Were the fits improved in character? Possibly, but not probably. Dr. Marshall Hall, in his lectures at the College of Physicians, allowed that a fit had followed very shortly after the operation, in which the tongue was bitten. A Mrs. Dwellie, living in the adjoining garret to the patient's, and who frequently went to the patient's assistance when she heard the noise and struggle of the fit, states explicitly that the convulsions were as frequent and violent, and the subsequent torpor as prolonged after the operation as before it.

A Mrs. Smith, also, an aunt of the patient, who had known her from childhood, and who saw her several times a week during the whole of her life, makes the same statement. Miss Lewis, on the contrary, who lives on the first floor of the house in the garret of which the patient lived, thinks the fits, after the operation, were not so severe or frequent as before it; but why she thinks so is not very evident. She saw her in but few fits, and in none (there is reason to believe) from the commencement. Indeed, it is to be understood that this witness was infirm and half crippled, and often quite an invalid; that she had to be fetched from the top of the house, and then to mount up two flights of stairs before she could get to the place where the patient was; so that the fit must have been far from its commencement before she could see it. The last fit, also, which was evidently of great violence, is spoken of only as 'apparently more severe than usual,' showing that the ordinary fits were severe; and the patient was 'expected to recover as usual,' showing that death occurred unexpectedly in what was regarded as an ordinary fit. Concerning the state of the general health, there are two opinions. Miss Lewis says this was better: Mrs. Dwellie and Mrs. Smith say there was no perceptible improvement until within two or three months of her death, fifteen or sixteen months after the operation. The cause of death is very obscure. It could not be, however, from the strangulation of laryngismus, for the inner tube was removed at the beginning of the last fit, as it was in all the fits in which the patient was watched. Indeed, there was never any neglect or mismanagement about the tube (which reflects the highest credit on Mr. Anderson's mechanical ingenuity), and the patient herself had so schooled herself to it that she could remove and cleanse it, and did so remove and cleanse it, many times a day. The fatty state of the heart, as Dr. Hall supposes, might have had something to do with death, for death happened shortly after the commencement of the seizure; but, on the other hand, it is not to be forgotten that there was stertorous breathing, blackness and turgescence of the head and neck, with distended sinuses, distinct cerebral puncta, and other signs showing that death might have been caused by coma."

To us these cases have almost a ludicrous aspect. The one corks his vent-hole, and, in spite of the theory, is henceforth free from fits; the other is most careful to keep her vent-hole free, and when she is unable to do this herself, others do it for her, and yet her fits continue as before, and forsooth, she must die in one—not a fit of syncopal epilepsy, as Dr. Hall would fain persuade himself and others, but a fit of genuine unmistakeable asphyxial epilepsy. How, with these cases to begin with, Dr. Hall should have continued to advocate his pet theory, passes our comprehension.

In the attack itself our conduct is expectant and precautionary—the endeavours should chiefly be directed to the prevention of physical injury, and to obviating the strong tendency to asphyxia; but we cannot approve of tracheotomy as a resource with this view. Any attempt at internal treatment is unsuccessful. Should the attack continue long, heat and stimulants may be applied to the epigastrium, abdomen, and legs. If there be premonitory signs, the fit may not unfrequently be prevented entirely, by the administration of an emetic, a purgative, or, as Dr. Radcliffe has observed, in some cases, by a stimulant, as a glass of wine. The choice must be made between those according to the circumstances of the individual case.

We regret that our limits compel us to be thus fragmentary in our hints on treatment. We leave the subject with a firm persuasion that much may still be done in this intractable disease, by a careful application of a rational system of treatment, founded upon the principles laid down—and with a full conviction that earnest observation and patient inquiry will, in this and all other sciences, compel nature to an answer, and ultimately force her to reward her faithful votaries by the revelation of her long-cherished secrets.

---

Since the preceding article has been in print we have received M. Delasiauve's recent treatise on Epilepsy, and this we will now proceed to notice in a sequel, rather than that our readers should not be at once acquainted with it.

The position occupied by this author, the numerous cases upon which his observations are founded, and the evident care and pains adopted to obtain correct deductions from accurate observations, make the statements contained in this work of great value—and even those from which we are compelled to dissent have their own peculiar worth in another respect, which we shall afterwards notice.

The first point worthy of specification is with regard to the *division* of epileptic attacks into the major and minor kinds. One author very properly and accurately defines the differences between the slightest possible kind (absence), the second (vertige), the third (accès intermédiaires), and the fourth (chutes—attaques ou accès complets). The symptoms are well drawn, but we think that it is an error to consider the last form alone as the type of the disease when confirmed and fully established. We have seen already, that in some cases (the number bearing a very appreciable relation to the whole) there are *only* the incomplete attacks or vertigoes; no convulsions, or only such as are very slight, occurring; and that these, so far from being, as our author terms them, but "shadows and abortions" of the full attack, are followed even more quickly and certainly than the convulsive attacks, by those systemic derangements and those mental degradations which are amongst the most fearful of the consequences of epilepsy. Dr. Delasiauve does not appear to recognise this as essentially true, yet as seen above, high authority is very positive on the point.

Our author lays much stress on the duration of the complete attack, limiting it to a minimum of two minutes and a maximum of five.

Our own experience is very much at variance with this, as we have known many instances where the attack has lasted for hours, even taking the indication of its termination, as pointed out in the work before us, at the moment when the respiration recovers its freedom. Two cases have come under our immediate care recently, which illustrate this point. One, which was epilepsy of eleven years' standing, had fits varying from one minute to several hours, and the final attack, which was fatal, lasted above twelve hours. In all these there was the most severe embarrassment of the respiration. In the second case, which still survives, the last attack which we witnessed lasted four hours and a half, the convulsions all this time being violent, and the respiration so very much impeded, that even the desperate resource of tracheotomy was entertained, though ultimately dispensed with.

The researches into the precursory symptoms are very valuable; our author finds them to be much more numerous than are generally supposed; in fact, that the attacks *with* premonitory signs are about one third more numerous than those occurring without any warning immediate or remote. In 264 carefully observed cases he found immediate precursory symptoms in 150 cases, and remote signs in 35. Another interesting point mentioned is, that occasionally these premonitions have been *objective* exclusively; that is, they have been unnoticed by the patient, but evident to the bystanders.

We have already given the very vague and varying statistics of these warning signs; we would urge this as a point especially deserving of attention on all who are interested in the study of this affection, inasmuch as we are convinced that close observation will *most frequently* detect some change, which may serve as a shadow of the coming event, and also that the recognition of such changes, and the instant adoption of such therapeutic or hygienic applications as they may seem to indicate, will be most important agents in the prevention and ultimate cure of the attacks, and most especially in those cases where we do not suspect organic mischief, but where *an evil habit* of system requires breaking off.

With regard to *periodicity*; it is recognised as feebly marked in some few cases, and in some others as indicating the type of intermittent fever, and then becoming a useful guide to treatment. The following table as to the recurrence of the fits is interesting:—

| | | |
|---|---|---|
| Fits almost daily in . . . . | 9 | cases |
| „ in from 2 to 6 days . . | 42 | „ |
| „ once a week . . . . . | 20 | „ |
| „ 10 to 15 days . . . . | 9 | „ |
| „ 15 to 30 days . . . . | 9 | „ |
| „ with very irregular intervals, | 21 | „ |
| | 110 | |

To make these statistics more complete, we add a table of the results obtained by M. Herpin, M. Beau, and M. Leuret, for convenience of comparison all reduced to the proportions of 100.

| | Herpin. | Beau. | Leuret. |
|---|---|---|---|
| One or more attacks daily in | 22 | 10 | 2 |
| One to six in the week „ | 36 | 25 | 32 |
| One to four in the month „ | 16 | 45 | 59 |
| One to eleven in the year „ | 26 | 20 | 7 |
| | 100 | 100 | 100 |

With regard to anatomical lesions, Dr. Delasiauve adds to our stock of observations, but, as he confesses, little to our knowledge of the disease derived from thence. In 95 cases he found important lesions in 43, equivocal lesions in 31, and a total absence of lesion in 21; and he concludes with regret that we do not yet know, nor even suspect the true seat of epilepsy.

But one of the most important points which we have to notice, is the hereditary transmission of epilepsy, in reference to which we find some extraordinary data in the work before us. We have seen above, that in a great majority of the instances tabularized by M. Herpin, there were indications of a family tendency either to epilepsy or to some disease of the nervous centres, or at least to some considerable functional disturbances.

Thus in 68 cases, slightly to recapitulate, he meets with 10 cases of ancestral epilepsy, 24 of mental alienation, 11 of apoplexy with hemiplegia, and 13 of chronic meningitis and hydrocephalus. We must, however, bear in mind that these were not all in separate individuals, and therefore the proportion of ancestral affections is not quite so large as from a cursory glance it would appear. Yet, even making every allowance, how different is this statement from the results indicated in the following table! Out of 300 cases there was

| | | |
|---|---|---|
| Absence of hereditary indications in . . . . . | 167 | cases |
| Positive declaration of non-existence of such indications in . . . . . . . . . . . . | 120 | „ |
| Existence of epilepsy in relations in . . . . | 5 | „ |
| Nervous and cerebral affections in . . . . | 6 | „ |
| | 300 | |

Perhaps this striking difference may be in part accounted for, by taking into consideration that these 300 cases were hospital patients, in whom, for obvious reasons, the difficulties attendant upon eliciting information are very much greater than in private practice. There is also an irresistible tendency to conceal facts bearing upon this question.

But making all possible allowances, we find most startling discrepancies in the writings of various authors on this branch of the subject—these and other differences we shall have again briefly to allude to in the sequel.

We do not find much additional light thrown upon the already discordant views of the influence of menstruation and pregnancy, on either the development or the progress of the disease. With regard to solitary vice, Dr. Delasiauve gives it a more prominent causative place than most authors—thus, MM. Bouchet and Casanvieille attribute epilepsy to this cause in 3 out of 77 cases; M. Beau, in only 1 in 273; M. Herpin in 1 in 27; Dr. Delasiauve gives 25 in 200 as the proportion M. Leuret alone exceeds this calculation, 12 in 67 being the relativ numbers. It must, however, be always impossible to calculate the precise influence which a vice so general has upon any given disease; but that it has a striking and powerful influence, by reducing the general powers, and by exaggerating irritable mobility, cannot be doubted.

With respect to treatment, we find nothing especially new; tonics appear to enjoy less favour, and sedatives somewhat more, than we have been inclined to accord to them. The effects of the preparations of copper are "isolated, slow, and suspicious;"—nitrate of silver almost useless—sulphate of quinine not answering the expectations formed of it. Valerian, assafœtida, artemisia, and camphor, are spoken very highly of, the latter being of use, chiefly indirectly, through its aphrodisaic action. Ammonia is of much service.

The earnest and sincere searcher after truth for its own sake, has no more distinguishing characteristic than that total and absolute self-negation, which ever and again leads him to distrust himself rather than nature, to relinquish theory rather than to neglect or warp fact and observation. When convicted of error, instead of mortification that he is wrong, he finds matter of congratulation and rejoicing that there is still another chance, by rigid observation and rectification of error to discover the truth of the secret which has so long eluded him. He perceives that still the mystery is *not demonstrated* to be unfathomable, since the *most* accurate means have not yet been taken to elucidate it. Such is the foundation of our hope, that yet we may have light shed upon the obscure subject of our essay. We have seen that the significance of symptoms is mistaken,—that the varieties of disease are doubtful,—that the pathological and anatomical conditions are involved in mystery,—that the causes are obscure in their operation and their efficiency,—that the prognosis is not agreed upon,—that the treatment is empirical. Yet we have hopes for a better state of things, for do we not see plainly that observation, accurate and untheorizing, is yet deficient? This is sufficiently answered by the varying accounts which are given by different authors of simple matters of fact. A better state of things *is* arising; observations, honest and sincere, *are* multiplying and accumulating, but "non tantum numerandæ sunt, sed etiam perpendendæ observationes," we must have facts well weighed and well observed, and in sufficient numbers—then, and then only, shall we be in condition aptly to interrogate nature as to her meaning.

We cannot better conclude our remarks than by the closing paragraph of M. Herpin's introductory chapter:—

"Of the hundred thousand physicians who practise their art in Europe, let but one tenth—one hundredth, devote but a minute fraction of their time, to observe conscientiously, to note exactly, and to review methodically, the results; * * * * and it will require comparatively but a few years to elevate a magnificent monument which neither the efforts of sectaries nor the wear of ages can injure. Each generation will add to it its own work, but it will respect that of its predecessors, and the edifice will gain in grandeur, and lose nothing of its solidity or harmony of proportion."

---

